# Strengthening the human rights framework to protect breastfeeding: a focus on CEDAW

**DOI:** 10.1186/s13006-015-0054-5

**Published:** 2015-11-18

**Authors:** Judith Galtry

**Affiliations:** Visiting Fellow, Regulatory Institutions Network (RegNet), Australian National University, Canberra, ACT Australia

**Keywords:** CEDAW, Breastfeeding

## Abstract

**Background:**

There have been recent calls for increased recognition of breastfeeding as a human right. The United Nations Convention on the Elimination of All Forms of Discrimination against Women, 1979 (CEDAW) is the core human rights treaty on women. CEDAW’s approach to breastfeeding is considered from an historical perspective. A comparison is drawn with breastfeeding protection previously outlined in the International Labour Organization’s Maternity Protection Convention, 1919 (ILO C3), and its 1952 revision (ILO C103), and subsequently, in the United Nations Convention on the Rights of the Child, 1989 (CRC).

**Discussion:**

Despite breastfeeding’s sex-specific significance to an international human rights treaty on women and CEDAW’s emphasis on facilitating women’s employment, CEDAW is, in reality, a relatively weak instrument for breastfeeding protection. In both its text and subsequent interpretations explicit recognition of breastfeeding is minimal or nonexistent. Explanations for this are proposed and contextualised in relation to various political, social and economic forces, especially those influencing notions of gender equality. During the mid to late 1970s -when CEDAW was formulated - breastfeeding posed a strategic challenge for key feminist goals, particularly those of equal employment opportunity, gender neutral childrearing policy and reproductive rights. Protective legislation aimed at working women had been rejected as outdated and oppressive. Moreover, the right of women to breastfeed was generally assumed, with choice over infant feeding practices often perceived as the right NOT to breastfeed. There was also little awareness or analysis of the various structural obstacles to breastfeeding’s practice, such as lack of workplace support, that undermine ‘choice’. Subsequent interpretations of CEDAW show that despite significant advances in scientific and epidemiological knowledge about breastfeeding's importance for short-term and long-term maternal health, breastfeeding continues to be inadequately addressed in international human rights law on women. A comparison is made with CRC and its subsequent elaborations. Increasing recognition of the need to protect, promote and support breastfeeding within the framework of CRC but not that of CEDAW suggests that breastfeeding is regarded primarily as a children's rights issue but only minimally as a women's rights issue.

**Summary:**

The human rights framework requires strengthening in every direction to protect, promote and support breastfeeding. Discussion is needed regarding whether a separate strengthening of the international human rights framework on women is required with regard to breastfeeding.

## Background

In recent decades there have been calls for greater recognition of breastfeeding as a human right [1–4]. Key relevant international treaties include the International Labour Organization’s *Maternity Protection Convention* (MPC), the *United Nations Convention on the Elimination of All Forms of Discrimination against Women*, 1979 (CEDAW) and the United Nations *Convention on the Rights of the Child,* 1989 (CRC).

This paper focuses on how CEDAW as the key human rights treaty on women addresses breastfeeding. CEDAW’s development is considered from an historical perspective. A comparison is drawn with breastfeeding protection outlined sixty years earlier, in 1919, in the International Labour Organization’s *Maternity Protection Convention* (ILO C3), and its 1952 revision (ILO C103), as well as in the United Nations *Convention on the Rights of the Child*, 1989 (CRC) adopted ten years after CEDAW.

Despite breastfeeding’s significance to an international legal framework on women and the fact that CEDAW’s expressed aim is to eliminate *all forms of discrimination* against women, explicit recognition of the need to protect breastfeeding is minimal in the text of CEDAW. Possible reasons for this minimisation are contextualised with regard to particular political, social and economic forces, especially those influencing notions of gender equality.

The way CEDAW has addressed breastfeeding since the time of its adoption by the United Nations in 1979 is then examined in relation to its subsequent interpretations. Even several decades after the Convention’s adoption, and despite significant international advocacy to assert breastfeeding as part of a women’s rights framework, breastfeeding continues to be inadequately addressed.

The approach to breastfeeding outlined in the *Convention on the Rights of the Child * (CRC), as well as in its subsequent elaborations, is referred to as a comparison. When comparing the two conventions and their subsequent interpretations, it appears that breastfeeding, by its placement and framing, has become increasingly seen as a human rights concern of relevance for children, but only minimally for women.

The paper concludes that the human rights framework requires strengthening in every direction to protect, promote and support breastfeeding. While there are various avenues to advancing breastfeeding on the women’s agenda, specific discussion is needed as to whether a separate strengthening of CEDAW is required with regard to breastfeeding. A potential solution is proposed.

### A protectionist approach: women, work & breastfeeding in the early twentieth century

The first international treaty to address breastfeeding was the International Labour Organization’s *Maternity Protection Convention* (MPC) (ILO C3) in 1919. (Table 1 for human rights instruments referred to).

**Table 1:** Various international policy instruments (binding & non-binding)

Year	Policy instruments (binding & non-binding)	Approach to breastfeeding
1919 (adopted by the International Labour Organization)	Maternity Protection Convention No. 3 [binding]	Provides for: maternity leave (six weeks prior to and six weeks after birth); cash and medical benefits; job protection while on maternity leave; and two half-hour nursing breaks during working hours.
1952 (revised by the International Labour Organization)	Maternity Protection Convention No. 103 [binding]	Provides for: at least 12 weeks of job-protected maternity leave; extension of leave for medical reasons; higher cash benefits through compulsory social insurance or public funds; and nursing breaks to be counted as working hours and paid.
1979 (adopted by the UN General Assembly) 1981 (Date in force)	Convention on the Elimination of All Forms of Discrimination against Women (CEDAW) [binding]	Requires governments to: “ensure to women appropriate services in connection with pregnancy, confinement and the post-natal period…as well as adequate nutrition during pregnancy and lactation”.
1981 (adopted by the World Health Assembly)	International Code of Marketing of Breast-Milk Substitutes [non-binding]	Recommends restrictions on the marketing of breastmilk substitutes, such as infant formula, to ensure that mothers are not discouraged from breastfeeding.
1989 (adopted by the UN General Assembly) 1990 (Date in force)	Convention on the Rights of the Child (CRC) [binding]	Requires governments to: “ensure that all segments of society, in particular parents and children, are informed, have access to education and are supported in the use of basic knowledge of child health and nutrition, and the advantages of breastfeeding”.
1990 (World Health Organization and UNICEF)	Innocenti Declaration on the Protection, Promotion and Support of Breastfeeding. Breastfeeding in the 1990s: A Global Initiative. [non-binding]	Outlines the need for the removal of obstacles to breastfeeding within the health system, the workplace and the wider community.
1991 (World Health Organization and UNICEF)	Baby-friendly Hospital Initiative (BFHI). [non-binding]	Outlines steps to protect, promote and support breastfeeding in hospital and maternity settings
1995	Beijing Declaration and Platform for Action (that came out of the Fourth World Conference on Women) [non-binding]	Identifies a range of measures to protect breastfeeding.
1999	CEDAW Committee issued an interpretation (adopted as a general recommendation) of CEDAW’s requirements relating to Women and Health (Article 12)	No mention of lactation/breastfeeding.
2000 (revised by the International Labour Organization) 2002 (Date in force)	Maternity Protection Convention No. 183 (& Recommendation No. 191) [binding]	Provides for: the right to one or more daily breaks or a daily reduction of hours of work to breastfeed; the number & duration of nursing breaks to be determined by national law and practice & paid as working hours. - the possibility (if practical & employer is agreeable) of combining daily nursing breaks to allow reduced hours of work at the beginning or end of the working day (Rec No. 191). - where practicable, the establishment of facilities for nursing under adequate hygienic conditions at or near the workplace (Rec No. 191).
2002 (endorsed by the World Health Assembly)	World Health Organization/UNICEF Global Strategy for Infant and Young Child Feeding [non-binding]	Outlines range of measures & operational targets for protecting, promoting and supporting breastfeeding.
2005	WHO/UNICEF Innocenti Declaration on the Protection, Promotion and Support of Breastfeeding [non-binding]	Affirms the targets of: the 1990 Innocenti Declaration and the 2002 Global Strategy for Infant and Young Child Feeding.
2013	CRC Committee formulated a General Comment on the right of the child to the enjoyment of the highest attainable standard of health	Requires State parties support: the global recommendation for 6 months exclusive breastfeeding alongside appropriate complementary foods preferably until two years of age; the baby-friendly hospital initiative; legislation based on the International Code on Marketing of Breastmilk Substitutes and relevant World Health Assembly resolutions, and requires that private companies comply with these. Also requires protection of breastfeeding in employment context, including compliance with the ILO’s Maternity protection Convention.
2013	CRC Committee formulated a General Comment on State obligations regarding the impact of the business sector on children’s rights	Requires that businesses comply with the International Code on Marketing of Breastmilk Substitutes and relevant World Health Assembly resolutions and governments create employment conditions which facilitate breastfeeding.

In the aftermath of World War 1, key objectives were to retain women in paid work after the war due to a severe male labour shortage; to reduce high infant and maternal morbidity and mortality rates, especially among women working in factories; to increase population growth after massive loss of life; to breed ‘strong stock’ for replenishing the military [5].

While this Convention has been revised twice since 1919, its basic principles and provisions still stand. Like other ILO instruments, the MPC (ILO C3) 1919 outlined minimal standards for governments in developing and implementing sound labour policies/legislation. It established the right of women working in industry and commerce to maternity protection through maternity leave; the right to cash and medical benefits; the prohibition of dismissal during maternity leave; and the right to daily breastfeeding breaks during working hours.

The MPC was first revised in 1952 (ILO C103) and then again in 2000 (ILO C183). These Conventions vary in their provisions, including with regard to breastfeeding breaks and facilities in the workplace and maternity leave [6].

### A shift to gender equality

In the decades following the adoption of the original MPC in 1919 there was a move away from a protective approach towards women workers towards one based on the principles of non-discrimination and equality with men. The protective employment legislation of the first half of the twentieth century was re-examined and, in some cases, abolished. This included the prohibition on women’s night work; restricted working hours for women; and women’s exclusion from various occupations on sex based grounds [5].

This change in emphasis was reflected in ILO policy, including the *Discrimination (Employment and Occupation) Convention*, 1958 (ILO C111), which recognised the right of workers not to be discriminated against on various grounds, including that of sex, and various “promotional” instruments, including the *Equal Remuneration Convention*, 1951 (ILO C100) and the *Declaration on Equality of Opportunity and Treatment for Women Workers*, 1975.

Although discriminatory measures against women were abandoned and gender neutral measures increasingly adopted, special protection afforded to women workers during pregnancy, postbirth recovery and breastfeeding continued to be seen as a ‘pre-condition’ for non-discrimination and equality of opportunity in employment in ILO policy [5]. In 1952, the revised MPC (ILO C103) stipulated that nursing breaks were to be paid, but that the decision regarding their number and duration be left to national laws and regulations. An accompanying Recommendation (ILO R95) recommended: that nursing breaks be extended to at least one-and-a-half hours per day and facilities for nursing or day care financed from public/social security sources.

### Breastfeeding in CEDAW

The idea for a single, comprehensive international human rights treaty addressing non-discrimination and the role of women was raised in the mid-1970s [7]. As part of the lead up to the World Conference of the International Women’s Year in 1975, the United Nations Commission on the Status of Women undertook responsibility for preparing an internationally binding instrument on women. In 1979, after several years in the drafting, CEDAW was adopted by the UN General Assembly and came into force in 1981.

Consisting of a preamble and 30 articles, the Convention sets out internationally accepted principles on the rights of women that are applicable to all women, establishes the legal principle of non-discrimination on the basis of sex and affirms sex equality as a goal [8].

With respect to women in paid employment, CEDAW states that: special measures “aimed at protecting maternity shall not be considered discriminatory” (Article 4.2); that women should be afforded, “the right to protection of health and safety in working conditions, including the safeguarding of the function of reproduction” (Article 11.1f), with maternity protection identified as a core component of this goal.

In many respects, CEDAW’s maternity provisions closely resemble those contained in the *Maternity Protection Convention* [9]. In particular, Articles 11 and 12 of CEDAW say that governments are to ban dismissal on the grounds of pregnancy or maternity leave; enact maternity leave with pay without loss of employment, seniority and social benefits; and promote the development of child care facilities to enable parents to combine family and work responsibilities.

Despite these measures, CEDAW fails to identify the specific components of maternity, only referring broadly to “the function of reproduction” [10]. This has implications for breastfeeding protection. Significantly, despite being the first human rights treaty to refer to breastfeeding or, more specifically, ‘lactation’, CEDAW does not recognise that this female function requires protection in the workplace, such as through provision for breastfeeding breaks and facilities. Nor does it include lactation/breastfeeding along with “pregnancy”, “maternity leave” and “marital status” as a prohibited ground for employment dismissal. Given that one of CEDAW’s key aims is to facilitate women’s labour market participation, this oversight is paradoxical, while also reinforcing the common assumption that breastfeeding is more appropriately a private sphere practice.

Moreover, given that protection for breastfeeding in the workplace, including daily nursing breaks, had been specifically recognised as an essential component of maternity protection by the ILO sixty years earlier in 1919 (and again in 1952 in the revised MPC 103), CEDAW’s failure to address breastfeeding as an important equality and employment concern for women seems intentional.

Nor is the need to protect breastfeeding against the onslaught of infant formula feeding practices in hospital maternity settings acknowledged in CEDAW, despite the high profile nature of these concerns during the late 1970s.

In fact, the only direct reference made to breastfeeding/lactation in CEDAW can be found in Article 12 which says that women should be provided with “appropriate services in connection with pregnancy, confinement and the post-natal period…as well as adequate nutrition during pregnancy and lactation.” (paragraph 2).

### Why is there minimal protection for breastfeeding in CEDAW?

There are a number of factors that might explain the apparent downplaying of breastfeeding in CEDAW. One hypothesis is that CEDAW was drafted during the late 1970s when the medical community was only just waking up to the potential dangers associated with artificial infant feeding, as evidenced by the convening of the joint WHO/UNICEF Meeting on Infant and Young Child Feeding in 1979; the same year that CEDAW was adopted [11].

However, undermining this hypothesis is that this high level, cross agency Meeting on Infant and Young Child Feeding in 1979 occurred *after* the launching of the international boycott against the Swiss-based Nestlé corporation on account of its aggressive infant formula marketing practices in developing countries. The Nestlé boycott which led to calls for an international Marketing Code, had been launched in the United States in 1977, two years prior to the UN’s adoption of CEDAW; at a time when CEDAW was still under deliberation. International concerns about infant formula marketing practices and the impact on breastfeeding and infant mortality rates had also been raised earlier, including in 1974 in the War on Want's *The Baby Killer;* in 1978 in prominent hearings by the United States Senate Health and Scientific Research Subcommittee chaired by Senator Edward Kennedy; as well as via efforts by the World Council of Churches[12, 13].

So although it seems unlikely that policy makers involved in formulating CEDAW were unaware of concerns highlighted by the highly publicised boycott, unethical infant formula marketing practices tended to be viewed primarily as a *public health* concern affecting mainly impoverished ‘Third World’ populations. This assumption is possibly also reflected in CEDAW’s only point of reference to breastfeeding i.e. its provision that lactating women have access to ‘adequate nutrition’. (At that time, maternal malnutrition also tended to be viewed as a contraindication to breastfeeding promotion in the context of developing countries). Moreover, it is only since this time that evidence has accumulated to any compelling extent about the maternal benefits of breastfeeding [14, 15].

It has also been theorised that while many of the principles outlined in earlier treaties and declarations relating to women were incorporated into CEDAW, these earlier instruments were imbued with the fundamental values of ‘Western feminism’ [16, 17]. By the 1970s mainstream liberal Western feminist goals included greater reproductive autonomy and self-determination by women; an emphasis on equal treatment in the workplace; and support for the principle of gender-neutral childrearing. The issue of breastfeeding potentially undermined each of these goals.

Firstly, from the 1970s, with the dramatic influx of women into the labour market in many advanced economies and, alongside this, calls for equal employment opportunities for women, feminist discussions focused on women’s “sameness to” rather than their “difference from” the then male worker norm [18]. For feminist policy makers and legislators, the historical focus on women’s “difference”, which had justified past protective legislation, had increasingly come to be seen as costly for women [19].

The move from a “protectionist” and “maternalist” philosophy toward one of equal employment opportunity and equal treatment often meant, in effect, that while the inescapable “difference” of pregnancy had to be taken into account in any equality and workplace framework, breastfeeding, by contrast, tended to be perceived as non-essential and optional [20, 21].

Secondly, at the strategic level, breastfeeding was inextricably entangled with traditional notions of women’s responsibility for childrearing and, linked to this, their confinement to the private sphere of household and family. An emphasis on breastfeeding potentially undermined a growing consensus on the importance of a gender neutral childrearing principle in family and employment policy [20, 22]. For example, Article 5 of CEDAW stresses the need to retain “an awareness of maternity as “a social function” while recognising that the care of children is “a shared responsibility between women and men” (paragraph 5b). This trend was also reflected in ILO policy by the replacement of the ILO’s 1965 *Recommendation 123 on the Employment of Women with Family Responsibilities* with, in 1981, the gender-neutral *Convention on Equal Opportunities and Equal Treatment for Men and Women Workers: Workers with Family Responsibilities* (ILO C156).

Both the commercialisation and professionalization of child care and the popular representation of infant formula as “liberation in a can” supported this conception of equality [23]. Further exacerbating these trends hostile to breastfeeding was (and still is) the widespread sexualisation of breasts [24, 25].

Lastly, while women’s employment was a central focus for liberal feminist policy makers during the 1970s, the international women’s movement was simultaneously organising around reproductive rights and autonomy for women, particularly their greater control over fertility. In 1979, at an international women’s workshop in Bangkok, the goals were stated thus: “First, the freedom from oppression for women involves not only equity, but also the right of women to freedom of choice, and the power to control their own lives within and outside of the home. Having control over our lives and our bodies is essential to ensure a sense of dignity and autonomy for every woman . . .” [26]. Women’s rights with regard to infant feeding were often construed as the right NOT to breastfeed [27]. It did not always occur to policymakers and legislators (as evidenced, for example, by the United States’ ‘equality versus difference’ debates [20]) that, the lack of structural provisions such as breastfeeding breaks in the workplace meant that many women did not actually have the right to breastfeed. There was little recognition that breastfeeding requires intense support at all levels of society [28]. It also was, and continues to be, commonly assumed that outside the developing country context lack of breastfeeding is safe, although United States research shows this to be a fallacy [29, 30].

Even a decade after CEDAW's adoption by the United Nations, various arguments were put forward in the ILO context in favour of a new maternity convention that weakened rather than strengthened support for working women on the grounds that: stronger support for maternity protection would work against greater employment opportunities for women; would be pronatalist, leading women in already overpopulated countries to have even more babies; and was impractical, given that in the climate of the time anything that increased the cost of labour (or reduced labour productivity) was being opposed by most governments [31].

### International policy developments on breastfeeding prior to CEDAW’s formation

The minimisation and, sometimes, even the failure to address breastfeeding in much gender-based policy during the pre-CEDAW years seems less surprising when one considers that even at the highest level of international health policy breastfeeding had been addressed only minimally. For while the World Health Organization formally dates from 1948, it was not until 1974 that a World Health Assembly resolution first contained the word “breastfeeding”, but with the focus uniquely on the health and development of children [32].

In 1978, a second resolution expanded on this by recommending implementation of appropriate measures, including: “legislative and social action to facilitate breastfeeding by working mothers”; “regulating inappropriate sales promotion of infant foods that can be used to replace breast milk”; and development of “a programme of research … in nutrition … aimed initially at the prevention of malnutrition in pregnant and lactating women and in young children by promoting adequate nutrition of the mother and by encouraging breastfeeding . . .” [33].

However, a subtle, yet significant, policy-framing shift was nevertheless detectable in the following year in the statement adopted by consensus during the Joint WHO/UNICEF Meeting on Infant and Young Child Feeding (Geneva, 9–12 October 1979), which declared that mothers and their infants “form a biological unit” with their nutritional and health status unable to be seen independently of each other, and that breastfeeding is best not only for the baby but also for the mother, “including the physical, emotional, and psychological aspects of her health” [34].

### Subsequent developments in relation to CEDAW

Since the time of CEDAW’s adoption in 1979, there have been various related developments with potential relevance to breastfeeding.

In 1999, the Committee on the Elimination of Discrimination against Women, which monitors the implementation of the Convention, issued an interpretation (adopted as a general recommendation) of CEDAW’s requirements relating to Article 12 concerning Women and Health. This states that:

States parties should report on their understanding of how policies and measures on health care address the health rights of women from the perspective of women’s needs and interests and how it addresses distinctive features and factors that differ for women in comparison to men, such as:

(a) Biological factors that differ for women in comparison with men, such as their menstrual cycle, their reproductive function and menopause . . . [35]

Noticeably, two decades after CEDAW’s adoption, and despite the identification and inclusion of every other female-specific process, there is no reference to lactation. At best, it may be assumed to be an aspect of women’s ‘reproductive function’. This lack of actual mention represents a step back even from the text of the Convention itself which, at least, refers to lactation, even if minimally.

Yet, by this time - the late 1990s - there was a growing body of research on the risks for maternal health of not breastfeeding [36], as well as an emergent emphasis on breastfeeding as a human right. The WHO/UNICEF *Innocenti Declaration on the Protection, Promotion and Support of Breastfeeding* had been signed in 1990 and was the first treaty, albeit non-binding, to assert breastfeeding as a ‘right’ of women, although the use of rights terminology was confined to the employment context [37]. Moreover, in 1995 the *Beijing Declaration and Platform for Action* (that came out of the Fourth World Conference on Women) had a strong focus on breastfeeding. It calls for governments, in collaboration with key national and international stakeholders: to promote breastfeeding; to fully implement the *International Code of Marketing of Breast-milk Substitutes* and to “enable mothers to breastfeed their infants by providing legal, economic, practical and emotional support” (paragraph 106(r)); to “eliminate discriminatory practices by employers and to take appropriate measures in consideration of women’s reproductive role and functions, such as the denial of employment and dismissal due to pregnancy or breastfeeding” (paragraph 165(c)); and to “promote the facilitation of breast-feeding for working mothers” (paragraph 179(c)). It also called for all countries to ratify CEDAW and the CRC. This successful focus on breastfeeding at Beijing was likely linked to intense lobbying efforts by prominent breastfeeding advocacy groups. Despite this emphasis on breastfeeding, the Beijing Declaration – like the WHO Code and the Innocenti Declaration – does not have the same legally binding status as CEDAW and CRC.

Despite this apparent underplaying or omission of breastfeeding in CEDAW’s text and its subsequent interpretations, concerns relating to breastfeeding protection, including in the workplace setting, have occasionally been raised by countries in their periodic reports to the CEDAW Committee. In its annual sessional reports, the Committee has sometimes recommended that particular governments increase breastfeeding protection and support.

For instance, in 1998 - one year prior to the adoption of its General Recommendation on Women and Health which, as shown, neglected to mention breastfeeding - the Committee on CEDAW noted in response to Panama’s report its ‘deep concern’ that women in that country had no effective protection with respect to maternity leave and breastfeeding breaks and recommended their ‘vigorous’ implementation [38]. The following year the Committee commended Algeria for its labour legislation which “contains specific provisions relating to maternity leave and breastfeeding breaks that protect women from discrimination because of their parental responsibilities” [39]. Later, in 2012, the CEDAW Committee commended the New Zealand government for “a number of positive legislative and policy reforms for the advancement of women, . . . including *The Employment Relations (Breaks, Infant Feeding, and Other Matters) Amendment Act* of 2008 which promotes breastfeeding in the workplace” [40].

### Breastfeeding & the Convention on the Rights of the Child (CRC)

A decade after the adoption of CEDAW, the United Nations Convention on the Rights of the Child (CRC) was adopted in 1989, and came into force in 1990. Like CEDAW, CRC is a legally binding treaty.

Various obligations of CRC are commonly cited as relevant to breastfeeding. These include: the child’s right: to life; to the highest attainable standards of health; to adequate nutritious food; the right of the mother to pre- and postnatal care; the rights of parents to measures assisting them in their work and parental responsibilities; as well as the best interests of the child being a primary consideration. However, the only direct reference to breastfeeding is in Article 24 which says that all of society, but particularly parents and children, should be “informed, have access to education and [be] supported in the use of basic knowledge of child health and nutrition, the advantages of breastfeeding . . .” (paragraph 2e).

Subsequent interpretations of CRC formulated by the Committee on the Rights of the Child, which oversees the Convention, have emphasised breastfeeding amid growing concerns about the burgeoning and uncontrolled global infant formula market [41, 42].

In 2013, the Committee formulated a General Comment on Article 24 concerning “the right of the child to the enjoyment of the highest attainable standard of health” [43]. Many aspects protective of breastfeeding are addressed, including support for: the global recommendation for 6 months exclusive breastfeeding alongside appropriate complementary foods preferably until two years of age; the baby-friendly hospital initiative; legislation based on the International Code on Marketing of Breastmilk Substitutes and relevant World Health Assembly resolutions, as well as the need for special measures to protect breastfeeding protection in the employment and childcare contexts, including compliance with the ILO’s Maternity Protection Convention 2000.

Also in 2013, the Committee issued a General Comment on State obligations regarding the impact of the business sector on children’s rights. Along with the requirement that businesses comply with the WHO Code, it identifies the need for governments to “create employment conditions within business enterprises which assist working parents and caregivers in fulfilling their responsibilities to children in their care such as: the introduction of family-friendly workplace policies, including parental leave; support and facilitate breastfeeding” [44].

In effect, there is greater emphasis on breastfeeding within the framework of CRC (both the Convention’s text and its subsequent interpretations) than that of CEDAW.

### The way forward

Recent developments with respect to breastfeeding in CRC are progressive. However, the question is raised as to why breastfeeding has not also been addressed more satisfactorily within the core human rights treaty on women, that of CEDAW.

Yet, despite its minimal focus on lactation, CEDAW is sometimes invoked as an important international treaty for providing guidance on protecting breastfeeding as a human right [45, 46]. The well intentioned aim is to provide women’s organisations in signatory countries with the means to approach their governments regarding their obligations in relation to breastfeeding.

In recent years, international discussions and forums have taken place regarding how to formulate a human rights approach to breastfeeding, including in relation to women and children [47, 48]. Following earlier iterations the following principle was formulated:

‘Infants have the right to be breastfed, in the sense that no one may interfere with their mothers’ right to breastfeed them’ [2].

This formulation is necessarily theoretical and aspirational because, in reality, the human rights of women in relation to breastfeeding are not *explicitly* recognised in any ratifiable and thus, legally binding international treaty, including, ironically, in the core human rights treaty specific to women. Unfortunately, in contrast to CEDAW and CRC, those policy instruments that do provide strong protection for breastfeeding, including the WHO Code, the Innocenti Declaration, the Beijing Platform for Action, and the Global Strategy for Infant and Young Child Feeding endorsed by the World Health Assembly in 2002, are non binding and thus do not have the force of international law behind them. Moreover, even in these instruments, the issue of women’s rights in relation to breastfeeding is explicit *only* in the Innocenti Declaration’s statement that governments enact “imaginative legislation protecting the breastfeeding rights of working women and established means for its enforcement”, also reiterated in the Global Strategy [49].

Despite its non-binding status, the joint World Health Organization/UNICEF Global Strategy for Infant and Young Child Feeding nevertheless represents a potentially important blueprint for strengthening international human rights law to address breastfeeding. It does not explicitly address breastfeeding as a specific right beyond the employment context. However, it unequivocally states that access to adequate nutrition and the highest attainable standard of health is a human rights issue which concerns both mothers and children together and its implementation plan includes policies directly supportive of breastfeeding which would make the realisation of these rights attainable.

Another avenue is to focus on the implementation of the 1995 *Beijing Declaration and Platform for Action* on gender equality and women’s rights. 2015 is the twentieth anniversary of the Beijing Declaration’s adoption (*Beijng Plus 20*). To mark this anniversary, the UN Commission on the Status of Women (CSW) convened a conference of world leaders and advocates to assess progress and remaining challenges for the Beijing Platform’s implementation. The Committee reiterated its undiminished “stature and significance as a roadmap for the achievement of gender equality… [which] continues to guide the global struggle against constraints and obstacles to the empowerment of women around the world” [50]. As part of this, various organisations prepared a combined statement calling on the Commission to include breastfeeding as a core component of the women’s agenda [51].

The implementation of the Beijing Platform’s breastfeeding related objectives seems an ideal avenue for advancing the recognition of breastfeeding as a human right of women. The recent global expansion of often uncontrolled infant formula markets makes this focus even more compelling [52].

## Conclusion

In recent years there has been an emphasis at both the international and national levels on breastfeeding as a human right. Among those core treaties which are commonly invoked as providing direct guidance regarding the advancement of breastfeeding on the international and national human rights agendas are the *International Labour Organization’s Maternity Protection Convention*, the *United Nations Convention on the Elimination of All Forms of Discrimination against Women* and the *United Nations Convention on the Rights of the Child*.

When comparing how breastfeeding is addressed in each of these conventions several factors stand out. First, despite CEDAW's focus on women, it addresses breastfeeding only minimally. Second, although one of CEDAW’s core aims is to facilitate women’s labour market advancement, it fails to identify the need for provisions to protect breastfeeding in the workplace, despite the precedent established much earlier in the ILO’s Maternity Protection Convention. Third, when CEDAW’s requirements are compared to those of CRC - including both their subsequent interpretations – it appears that breastfeeding, by its placement, is represented as a human rights issue mainly of relevance to children.

Although CEDAW is the core international human rights treaty on women with the expressed aim of countering *all forms of discrimination against women*, it provides, in reality, only weak protection for the female specific function of lactation. This minimisation of breastfeeding has both symbolic and real significance, given that the Convention and its elaborations guide member states in interpreting their obligations with respect to women.

CEDAW is the product of a very different era. Where breastfeeding specifically is concerned, CEDAW is outdated in the light of rapidly accelerating collective awareness. It is time for international human rights law to catch up with compelling scientific and epidemiological knowledge and cumulative advances demonstrating the significance of breastfeeding for both the short- and long-term health and well-being of women.

The human rights framework requires strengthening in every direction to protect, promote and support breastfeeding. It needs to be considered whether a separate strengthening of CEDAW is required with regard to breastfeeding. Any further interpretation in CEDAW of non-discrimination in relation to ‘maternity’ or ‘women’s reproductive function’ could also specify ‘pregnancy, lactation and post birth recovery’, as in ‘special measures aimed at protecting “maternity (pregnancy, lactation and post birth recovery)” or, alternatively’, ‘special measures aimed at protecting “women’s reproductive function (pregnancy, lactation and post birth recovery)” shall not be considered discriminatory’. This would have both symbolic and real significance in clearly and unambivalently asserting breastfeeding as a human right of women.

## Abbreviations

CEDAW: United Nations Convention on the Elimination of All Forms of Discrimination against Women
WHO Code: International Code of Marketing of Breast-Milk Substitutes
CRC: United Nations Convention on the Rights of the Child
ILO: International Labour Organization
MPC: Maternity Protection Convention
